# Protective Effects of (*E*)-β-Caryophyllene (BCP) in Chronic Inflammation

**DOI:** 10.3390/nu12113273

**Published:** 2020-10-26

**Authors:** Rosaria Scandiffio, Federica Geddo, Erika Cottone, Giulia Querio, Susanna Antoniotti, Maria Pia Gallo, Massimo E. Maffei, Patrizia Bovolin

**Affiliations:** 1 Department of Life Sciences and Systems Biology, University of Turin, Via Accademia Albertina 13, 10123 Turin, Italy; rosaria.scandiffio@unito.it (R.S.); federica.geddo@unito.it (F.G.); erika.cottone@unito.it (E.C.); giulia.querio@unito.it (G.Q.); susanna.antoniotti@unito.it (S.A.); mariapia.gallo@unito.it (M.P.G.); 2 Plant Physiology Unit, Department of Life Sciences and Systems Biology, University of Turin, Via Quarello 15/a, 10135 Turin, Italy; massimo.maffei@unito.it

**Keywords:** (*E*)-β-caryophyllene, biosynthesis and distribution, inflammation, metabolic disorders, obesity, steatosis, type II diabetes, cardiovascular disorders, pain, neurodegenerative diseases

## Abstract

(*E*)-β-caryophyllene (BCP) is a bicyclic sesquiterpene widely distributed in the plant kingdom, where it contributes a unique aroma to essential oils and has a pivotal role in the survival and evolution of higher plants. Recent studies provided evidence for protective roles of BCP in animal cells, highlighting its possible use as a novel therapeutic tool. Experimental results show the ability of BCP to reduce pro-inflammatory mediators such as tumor necrosis factor-alfa (TNF-α), interleukin-1β (IL-1β), interleukin-6 (IL-6), nuclear factor kappa-light-chain-enhancer of activated B cells (NF-κB), thus ameliorating chronic pathologies characterized by inflammation and oxidative stress, in particular metabolic and neurological diseases. Through the binding to CB2 cannabinoid receptors and the interaction with members of the family of peroxisome proliferator-activated receptors (PPARs), BCP shows beneficial effects on obesity, non-alcoholic fatty liver disease/nonalcoholic steatohepatitis (NAFLD/NASH) liver diseases, diabetes, cardiovascular diseases, pain and other nervous system disorders. This review describes the current knowledge on the biosynthesis and natural sources of BCP, and reviews its role and mechanisms of action in different inflammation-related metabolic and neurologic disorders.

## 1. Introduction

The scientific interest for natural compounds as novel potential drugs has increased exponentially in the last few years, along with the number of trials and studies on nutraceuticals and herbal extracts, aimed to test their effects on many disorders, including obesity, type II diabetes (T2D), cardiovascular disease (CVD), NAFLD and also cancer [1,2,3,4].

The sesquiterpene hydrocarbon (*E*)-β-caryophyllene (BCP) is one of the most studied and promising natural compounds [5,6,7,8,9,10]. In recent years, modulatory and pharmacological effects of BCP have been demonstrated in numerous organs such as liver [11], kidney [12] and brain [13]. BCP has been reported to exert therapeutic effects as antioxidant [11], anti-inflammatory [14] and anticancer [12,15]. Importantly, BCP has been identified as a fully selective agonist of CB2 cannabinoid receptors [16], one of the key members of the endocannabinoid system (ECS). The ECS is an endogenous system exerting regulatory control on food intake, metabolism and storage of calories and for this reason it represents a potential pharmacotherapeutic target for a wide range of metabolic disorders such as obesity, dyslipidemia, steatosis, diabetes and eating disorders [17]. The ECS is also involved in the regulation of inflammation [18] and in the modulation of depression, schizophrenia and chronic pain [6,19,20,21]. The selectivity of BCP for CB2 receptors avoids potential psychotropic effects mediated by brain CB1 cannabinoid receptor, being CB2 receptors mainly expressed in peripheral tissues and in central nervous system (CNS) immune cells [5,16]. Apart from CB2 receptors, BCP has been recently demonstrated to interact with members of the family of peroxisome proliferator-activated receptors (PPARs), in particular PPARα and γ, transcriptional factors belonging to the ligand-activated nuclear receptor superfamily [10,22,23].

This review will first present the molecular features, biosynthesis and distribution of BCP in plants, followed by current information on the molecular targets of BCP in animal cells. Finally, the beneficial effects of BCP on human health will be discussed; since many of them have been already extensively reviewed [5,6,7,9,24,25], this review will specifically focus on BCP effects in metabolic and neurological diseases/disorders, with special emphasis on those characterized by chronic inflammation.

## 2. Plant Distribution, Biosynthesis and Molecular Biology of BCP

BCP, a bicyclic sesquiterpene, is widely distributed in the plant kingdom. It is a secondary metabolite belonging to the macro group of terpenes, it exerts a pivotal role in the survival and evolution of higher plants and contributes to the unique aroma of essential oils extracted from numerous species [26]. BCP is one of the most widespread sesquiterpenes in floral volatiles, occurring in more than 50% of angiosperm families [27]. A recent study identified several plants able to produce high percentages and high yields of BCP [28]. The work, out of more than 300 selected species, identified top species like *Copaifera langsdforffii*, *Cananga odorata*, *Humulus lupulus*, *Piper nigrum* and *Syzygium aromaticum*, which provide a high percentage of BCP along with interesting essential oil yields. These species possess a high potential for BCP utilization; however, only a skillful molecular fractionation of the essential oil allows the removal of undesired or even toxic terpenes that sometimes may be present along with BCP [28]. For instance, essential oils from *S. aromaticum* (also known as clove oil) may contain relatively high percentages of the toxic compound eugenol. This compound may form eugenol–quinone methides in hepatocytes, which are responsible for the cytotoxicity mediated by eugenol; finally, methyl eugenol, a derivative of eugenol, is also hepatotoxic [29]. With the due fractionation, the high percentages of BCP provided by these plants can be used for the preparation of new drugs or dietary supplements aimed to improve health, prevent lifestyle illnesses and act as a valid support for chronical diseases such as pain, metabolic and neurological disorders [28].

Due to the wide variety of plants producing BCP, the chemical synthesis of BCP appears to be an inconvenient strategy. For instance, a chemical method for BCP synthesis required eight steps including reduction, dehydration by Mitsunobu activation, diastereoselective reduction, selective tosylation, deprotonation, carbonyl-forming elimination, desilation and wittig methylenation [30]. Another way of producing BCP could be through microbial fermentation, because microorganisms grow rapidly. Although significant BCP yields have been obtained by employing a multi-step metabolic engineering strategy to increase precursor and cofactor supplies for BCP production [31], the costs for biotechnological applications are still too high when compared to essential oil costs and yields.

Thus, plants remain the main factories for BCP synthesis. In plants, terpenes are synthesized by terpene synthases (TPSs) which accept the ubiquitous prenyl diphosphates geranyl diphosphate (GPP), farnesyl diphosphate (FPP) and geranylgeranyl diphosphate (GGPP) as substrates and convert them into the different mono-, sesqui- and diterpene skeletons, respectively [32]. In the biochemical pathway that leads to BCP, the five-carbon building blocks isopentenyl diphosphate (IPP) and its allylic isomer dimethylallyl diphosphate (DMAPP) originate from two alternative pathways: the cytosolic mevalonate (MVA) pathway and the plastidial methylerythritol phosphate (MEP) pathway [32]. However, there is no compartmental separation of the two pathways and the extent of this cross-talk depends on the species and the physiological conditions [33].

**Figure 1:**
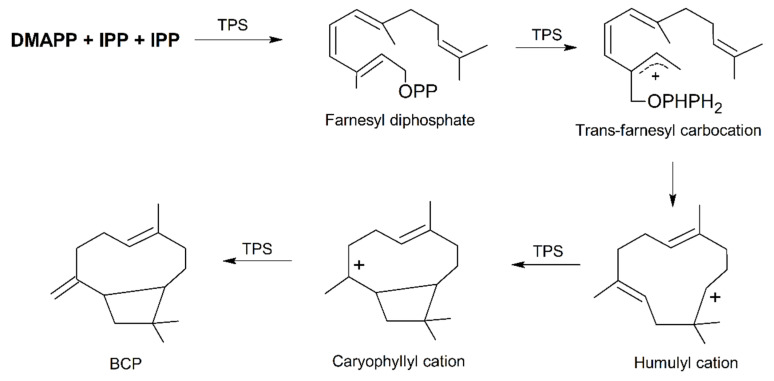
Simplified mechanism of the formation of (*E*)-β-caryophyllene (BCP) from DMAPP and IPP. Abbreviations: DMAPP, dimethylallyl diphosphate; IPP, isopentenyl diphosphate; TPS, terpene synthase. Modified from [36].

The condensation of one DMAPP and two IPP molecules catalyzed by farnesyl diphosphate synthase (FPPS) leads to the formation of farnesyl diphosphate (FPP) in the cytosol [34]. FPP serves as substrate for TPSs for synthesizing BCP and the reaction starts with FPP ionization to a *trans*-farnesyl cation [35]. This is then followed by a series of complex chemical mechanisms involving isomerizations, cyclizations, and rearrangements catalyzed by TPSs, which generate humulyl cation, caryophyllyl cation and eventually BCP [36] (Figure 1).

The BCP synthase (BCS) gene is characterized by the GVYXEP consensus sequence common to angiosperm sesquiterpene synthases [37], a conserved aspartate-rich region (DDxxD), which is crucial for the substrate binding [38] and by the xDx6E motif for metal cofactor binding [39]. A RR(X)_8_W motif, which is present in the *N*-terminal region and downstream of the *N*-terminal transit peptide, is assumed to participate in the ionization of the substrate [40] and is characteristic of the majority of the members of the terpene synthase subfamilies TPS-a and TPS-b [37]. Another conserved region, the RxR motif, is located 35 amino acids upstream of the DDxxD motif and is known to form the complex of diphosphate group after substrate ionization [41].

The role of BCP in plants is directly connected to plant defense and attraction. BCP is the main product of *Gossypium hirsutum* terpene synthase 1 (GhTPS1) and the expression of *GhTPS1* is induced in leaves of methyl jasmonate (MeJA)-treated cotton plants [42,43]. In *Medicago truncatula*, the MEP pathway-derived BCP is the main product of the terpene synthase 1 (MtTPS1); in this case the gene is induced by jasmonate (JA) and by the combination of JA and the ethylene precursor 1-aminocyclopropane-1-carboxylic acid (ACC) [44].

In grapevine five genes are known to code for BCP synthases [45] but only one (*VvGwECar2*) is actually expressed in all plant tissues and therefore accounts for most of the volatile production in vegetative parts and berries [46]. In this plant, flowers express two other genes (*VvGwECar1* and *VvPNECar2*) that seem to play an important role in these organs [47]. Biochemical analysis of *Oryza sativa* terpene synthase 1 (OryzaTPS1), coded by a rice terpene synthase gene involved in indirect defense against insects, showed that the enzyme functions as a BCS [48].

A partial cDNA for the BCP synthase gene (*MmCS*) was isolated from the expressed sequence tag (EST) library of *Mikania micrantha* leaves. *MmCS* expression was significantly increased in *M. micrantha* leaves within 3-days after wounding [49] and was found to be induced by high CO_2_ levels [50]. The Japanese pepper (*Zanthoxylum piperitum*) produces BCP in the secretory cavities. In this plant, the BCS ZpTPS1 specifically accepts the substrate FPP and is responsible for the biosynthesis of BCP [51]. In the flowers of the model plant *Arabidopsis thaliana*, the expression of terpene synthases can be induced by the phytohormones gibberellin (GA) and JA, and their induction increases the expression of *TPS21,* which encodes an enzyme that converts farnesyl diphosphate into BCP [52,53]. *TPS21* overexpression also demonstrated that BCP served as a defense against pathogens that invade floral tissues [35]. In *Liquidambar formosana*, the characterization of the BCS *LfTPS04* showed that seasonal differences in BCP content were correlated to the sesquiterpene synthase gene expression [54]. Expression patterns of BCP synthase gene during the development of *Artemisia annua* were observed in response to wounding and elicitation [55], whereas in *Pinus sylvestris* (Scots pine), insect oviposition enhances the transcription of the BCP/α-humulene synthase (*PsTPS1*), which in turn induces the attraction of an insect parasitoid [56]. Finally, in black pepper (*Piper nigrum*), *PnTPS1* produced BCP as a main product and α-humulene as a minor compound. The transcript level of *PnTPS1* correlated with the predominant BCP biosynthesis in black pepper, defining it as a relevant source of BCP [57].

## 3. Molecular Targets of BCP Action in Animal Cells

Apart from the functions of BCP in plants, recent studies outlined also a role of BCP in animal cells, highlighting its possible use as a novel therapeutic tool. Although the mechanism of action is not yet fully understood, studies indicate that BCP could act in animal cells through the specific binding to the CB2 receptor, a member of the endocannabinoid system [16], as well as the activation of PPARs, in particular PPARα and γ [10,22,23].

In the next few paragraphs, we will therefore describe more in detail these two classes of receptors.

### 3.1. CB2 Receptors

The cannabinoid receptors CB2 and CB1 belong to the endocannabinoid system, along with their natural ligands, the endocannabinoids, e.g., anandamide (AEA) and 2-arachidonoylglycerol (2-AG), and a plethora of enzymes involved in their biosynthesis and inactivation [58,59]. Nevertheless, exogenous cannabinoids do exist, the most potent of which is Δ^9^-tetrahydrocannabinol (THC), a well-known terpenoid present in *Cannabis sativa* var. *indica*, responsible for the psychoactive effects of marijuana [60]. The endocannabinoid system shares mediators and overlaps with metabolic processes of other signaling pathways; thus, a wider endocannabinoid-related network has been identified as “expanded endocannabinoid system” or “endocannabinoidome” [61].

Both CB1 and CB2 receptors are G-protein coupled receptors, with an extracellular *N*-terminal domain, seven transmembrane alpha-helices and an intracellular C-terminus [62,63]. They show 44% overall amino acid similarity and 68% homology in the transmembrane domain [63]; one of the most different regions is the one located in the extracellular domain, which is responsible for cannabinoid binding [64]. Both receptors signal through G_i/o_ proteins, thus they can inhibit adenylyl cyclase and activate mitogen-activated protein kinases (MAPKs). Worthy of note is the fact that MAPKs could regulate the activation of PPARs via direct phosphorylation. Differently from CB2 receptor, CB1-coupled G_i/o_ proteins can mediate activation of A-type and inwardly rectifying potassium channels, and inhibition of N- and P/Q-type calcium currents; in addition, CB1 receptors can signal through G_s_ proteins [65].

CB1 is the most abundant and widespread G-protein coupled receptor in the mammalian brain, being highly expressed by presynaptic termini of neurons in the cortex, amygdala, hippocampus, basal ganglia, and cerebellum, where its activation modulates neurotransmitter release [66]. Notably, CB1 is present also in many peripheral sites, including spleen, lung, thymus, heart [67].

On the other hand, CB2 receptors, are mostly distributed peripherally, in the cells of the immune system [68] and indeed the main role of CB2 seems to be immune modulation. However, recent studies showed low levels of CB2 also in the central nervous system [69], especially in microglial cells and its activation in association with neurodegenerative disorders [70].

The endocannabinoid system, due to its wide distribution, regulates various physiological functions, such as neurogenesis and neurodegeneration, cognitive and mood regulation, appetite and metabolism, muscle contractility, inflammation and immune functions [67].

In 2008, Gertsch and colleagues [16] showed that BCP is able to elicit some of its effects by acting as a fully functional agonist of the CB2 receptor. Notably, BCP binds selectively CB2 receptors, since it lacks significant binding activity to the human CB1 (hCB1) receptor, and it is unable to displace high-affinity ligands from hCB1. BCP was shown to bind hCB2 with an inhibitory constant K_i_ of 155 ± 4 nM, a binding affinity about 150 times lower than the potent high affinity cannabinoid ligand WIN55,212-2 (whose K_i_ for hCB2 is 1.2 nM). BCP, likely in its bioactive ββ conformation, binds to the hydrophobic region of the amphipathic hCB2 receptor binding pocket, being the putative binding site located adjacent to helices III, V, VI, and VII at the near extracellular site of the seven transmembrane domain. BCP acts as a full CB2 receptor ligand, since its binding activates CB2-mediated intracellular signaling, e.g., adenylate cyclase inhibition, intracellular calcium release and mitogen-activated kinases Erk1/2 and p38 activation [16]. Notably, BCP was shown to lead to anti-inflammatory effects, inhibiting lipopolysaccharide (LPS)-induced TNF and IL-1β expression in peripheral blood and attenuating LPS-stimulated Erk1/2 and JNK1/2 phosphorylation in monocytes. CB2-mediated BCP anti-inflammatory effects were also observed in an in vivo model, where BCP (5 and 10 mg/kg body weight), orally administered 1 h before carrageenan treatment, strongly reduces inflammatory response in wild-type mice but not in CB2 deficient mice [16].

Recent studies have demonstrated a role of BCP, through the activation of CB2 receptors, in the modulation of different processes. A specific BCP-mediated CB2 receptor activation has been demonstrated to be at the base for example of tumor suppression in glioblastoma where it has anti-proliferative effects and plays an anti-inflammatory activity through the modulation of NF-κB and PPARγ [71]. In LPS-induced interstitial cystitis in mice, the intravesical instillation or the oral treatment with BCP (100 mg/kg) resulted in a significant decrease in the number of adherent leukocytes, thus confirming an anti-inflammatory activity in bladder inflammation [72]. In another study [73], BCP (25 mg/kg) was able to prevent nucleoside reverse transcriptase inhibitors (NRTI)-induced neuropathic pain in mice, in a CB2 cannabinoid receptor-dependent manner; also, BCP treatment prevented the induced upregulation of inflammatory cytokines mRNA transcripts (i.e., Interferon γ, IL-6β and TNFα).

### 3.2. PPARs

PPARs are transcriptional factors belonging to the ligand-activated nuclear receptor superfamily involved in both metabolic and inflammatory responses (recently reviewed by Hong et al. [74]). The existence of receptors that could mediate peroxisome proliferation was first hypothesized in 1983 by Lalwani et al. [75]. PPARα (also called NR1C1) was later identified [76] as a new member of the steroid hormone receptor superfamily, that could be activated by different molecules, such as fatty acids and fibrates, a widely used class of hypolipidemic drugs. Further on, other members of PPARs family were discovered [77], namely PPARβ/δ (NR1C2) and PPARγ (NR1C3). PPARβ/δ is activated by saturated and polyunsaturated fatty acids and eicosanoids, as well as synthetic ligands. PPARγ is instead a specific receptor for thiazolidinediones (TZDs), such as troglitazone, rosiglitazone and pioglitazone, widely used for T2D treatment [74].

Although PPARs share high structural homologies, they are encoded by different genes, have different ligands, are expressed in different tissues and regulate different biological processes [74].

PPARα is abundantly expressed in the liver, where it acts as the master regulator of hepatic lipid metabolism [10,78,79]. PPARα is also present in the brown adipose tissue, heart, kidney and muscles [80]. PPARβ/δ is expressed in skeletal muscle, heart, gastrointestinal tract, adipose tissue, where it regulates fatty acid metabolism [81]. PPARγ is actually considered the master gene of adipogenesis, being mostly expressed in white and brown adipose tissue, but also the large intestine and the spleen [10,82]. Two major PPARγ isoforms, derived from alternative promoter usage, have been described. While PPARγ1 is expressed in many different tissues, PPARγ2 is specifically expressed in adipose tissue, although it can be induced in other districts by a high-fat diet [83,84]. PPARγ participates into the programmed differentiation of adipocytes by enhancing the 5’-adenosine monophosphate-activated protein kinase (AMPK) activity, a master energy sensor which regulates diverse metabolic pathways, increases mitochondrial activity and biogenesis in muscles and is responsible for the inhibition of adipogenesis [9,85]; moreover, PPARγ is an insulin sensitizer also involved in glucose homeostasis [74].

PPARs are ligand-activated nuclear receptors, that enhance the transcription of specific genes. They are characterized by 13 helices and a small four beta-sheets with a large hydrophobic binding pocket. Ligand binding induces a conformational change of the ligand-binding region and allow PPARs to form heterodimers with the retinoid-X-receptor (RXR); after activation, PPAR-RXR heterodimers can bind to specific DNA sequences (PPAR response elements, PPREs), which in turn stimulate the transcription of target genes [86]. The function of PPARs can be stimulated by the presence of specific coactivators or inhibited by corepressors, depending on the different tissues [87].

BCP has been demonstrated to interact with and to up-regulate members of the PPARs family. In particular, it can activate PPARα through a direct interaction with the ligand-binding pocket, thus regulating lipid metabolism [23].

Furthermore, studies indicate the triggering of PPARγ via a BCP-mediated CB2 receptor activation [88,89]. In this respect, PPARγ was demonstrated to be involved in BCP-dependent neuroprotection [90] and tumor suppression functions [71], as well as hypolipidemic effects and vascular inflammation amelioration [10], anxiolytic, anti-oxidant, anti-arthritic and anti-inflammatory effects [22,48,91].

## 4. Protective Effects of BCP on Metabolic and Neural Disorders Characterized by Inflammatory States

Recently, a growing number of studies has described multiple protective effects of BCP in several metabolic and neural disorders. Notably, these disorders are mostly characterized by chronic inflammation. In the next subsections, first the general features of chronic inflammation will be presented, followed by description of the protective and anti-inflammatory effects exerted by BCP in specific metabolic and neurologic diseases.

### 4.1. Chronic Inflammation as a Common Theme of Many Metabolic and Neurological Disorders

During the last decades, the complex phenomenon of inflammation has been extensively studied, and it has become clear that this condition is a fundamental feature of metabolic disorders such as obesity, type II diabetes (T2D), nonalcoholic steatohepatitis (NASH), NAFLD [92,93,94,95] and also neurological disorders, e.g., chronic pain, Parkinson’s and Alzheimer’s diseases [21,96,97].

The process of inflammation constitutes the tissue response to injury, and can be divided into acute and chronic inflammation. The first one is characterized by increased blood flow and vascular permeability, along with accumulation of inflammatory mediators such as cytokines, and immune cells, like neutrophils. The resolution of acute inflammation occurs rapidly [98]. Chronic inflammation involves progressive changes in inflammatory cells and the coexistence of tissue destruction and repair; it can become pathological because of the loss of tolerance or regulatory processes. Obesity and metabolic syndrome lead to an inflammatory state that differ from the classical response, being the inflammatory process systemic and characterized by a chronic low-intensity reaction [99]. During the first stages of white adipose tissue expansion, typical of an overweight condition, a set of acute pro-inflammatory mediators is required to support the remodeling of healthy adipose depots; however, if lipid accumulation progresses and the pro-inflammatory molecules persist, a state of chronic low-level inflammation is established, a typical scenario of an obesity condition. Recent studies showed that local infiltration of immune cells and enhanced production of pro-inflammatory cytokines lead to a condition that could generate insulin resistance, defective insulin secretion, and disruption of other aspects of energy homeostasis [93,94,100]. This chronic, low-grade, metabolically triggered inflammation is also called metaflammation, a condition mainly generated by metabolic excess and occurring in several metabolic tissues, including adipose tissue, pancreas, liver, muscle, brain, and heart [101,102,103].

The chronic inflammatory response develops from the interaction between adipose tissue resident immune cells, including macrophages, and the immune system, skewed to a proinflammatory phenotype. Under physiological conditions, immune cells coordinately regulate tissue integrity and metabolism by controlling the activity of subsets of T lymphocytes. These cells release a cascade of cytokines that regulate other immune cells such as mast cells, eosinophils and others, maintaining resident macrophages in a M2-polarized phenotype, therefore acting toward an anti-inflammatory and immunoregulatory direction [94,104].

Conversely, under pathological conditions like obesity, fatty liver disease and T2D, immune cells withstand changes such as the recruitment of M1-polarized macrophages, which display a more proinflammatory phenotype and secrete proinflammatory cytokines such as TNF-α,IL-1, IL-6, IL-12 and C-reactive protein [94,105,106,107].

The hypothesis that inflammation is linked to metabolic conditions was formulated in 1993 with the publication by Hotamisligil and colleagues, demonstrating that adipocytes constitutively express TNF-α, whose expression is noticeably increased in adipocytes of obese animal models (ob/ob mouse, db/db mouse and fa/fa Zucker rat) [108,109]. Nowadays, almost 30 years later, the link between obesity, diabetes and chronic inflammation has been confirmed [93,109] and it has been also proved that mice lacking functional TNF-α are more insulin sensitive and glucose tolerant than wild type animals [106,110]. Moreover, TNF-α is overexpressed in the adipose and muscle tissues of obese humans, and its exogenous dragging leads to insulin resistance [92,111].

Metabolic, inflammatory and innate immune processes are also regulated by lipids [92,112] through the action of PPARs and liver X receptor (LXR) families. Activation of these transcription factors inhibits the expression of several genes involved in the inflammatory response in macrophages and adipocytes, therefore suppressing the production of proinflammatory cytokines [92,113,114].

In this complex scenario, the Toll-Like receptor (TLRs) pathway plays a crucial role; in fact, it has been demonstrated that fatty acids may also bind to Toll-like receptors, inducing the synthesis of inflammatory markers in macrophages and exacerbating insulin resistance [107,115]. TLRs are a family of transmembrane receptors that recognize a variety of pathogen-associated molecular patterns (PAMPs), playing a fundamental role in the innate immune system. Presumably reflecting structural similarity between microbial and host membrane lipids, the subfamily of TLR2 and TLR4 have been reported also to recognize select host lipids and to play important roles in the pathogenesis of insulin resistance and obesity [99,115].

Hypertrophic obesity leads to a dysregulated subcutaneous adipose tissue and the accumulation of ectopic fat in many depots, such as in liver, where inflammation and hepatocyte injury are the hallmarks of NASH and NAFLD [95,116]. The innate immune system plays a fundamental role also in triggering and amplifying hepatic inflammation in liver lipid disorders. Activation of the inflammatory response pathway results from abnormal accumulation of lipids and consequent lipotoxicity [95]. It has been demonstrated that PPARα plays central role in this context, exerting anti-inflammatory activities in murine models of systemic inflammation. In fact, PPARα agonists specifically attenuate IL-6 concentration *in vitro* and *in vivo* [117,118].

Inflammatory reaction can also occur in chronic nervous system diseases, being neuroinflammation a central pathological feature of several neurological disorders such as Alzheimer’s disease, Parkinson’s disease and multiple sclerosis. Acting as a mediator in neurodegenerative pathologies, neuroinflammation causes microglia activation, mitochondrial dysfunction, as well as release of pro-inflammatory cytokines and reactive oxygen species (ROS) production [119].

### 4.2. Protective Effects of BCP on Metabolic Disorders Characterized by Chronic Inflammation

The current increase in metabolic diseases such as obesity, T2D, liver lipid disorders, and the complex framework of metabolic syndrome (MetS) is now considered a sort of epidemics caused by multiple factors, including loss of exercise, aging, wrong diet, genetic background and exposure to endocrine disruptors [120,121,122]. Most single metabolic diseases and complex syndromes such as MetS are characterized by inflammation, often becoming chronic and leading to CVD [123,124,125].

To cope with this global complex problem, several strategies are needed. Thanks to the growing data in this field, natural compounds are becoming an important approach to promote health and ameliorate metabolic disorder conditions [1,123,126,127,128,129,130].

Recently, a growing number of reports has described multiple protective effects of BCP in several metabolic disorders (see Table 1). In particular, BCP has been shown to actively promote the inhibition of lipid accumulation, fatty acids oxidation, decrease of visceral fat index, reduction of total cholesterol, triglycerides, and low density lipoprotein (LDL) cholesterol levels, decrease of hepatic 3-hydroxy-3-methylglutayl coenzyme A (HMG-CoA) reductase activity, reduction of body weight in animal models and action as insulinotropic agent [5,10,78,126,131,132,133,134,135,136]. Notably, the effectiveness of BCP as anti-inflammatory agent is at least partly due to its ability to inhibit the main inflammatory mediators, e.g., inducible nitric oxide synthase (iNOS), IL-1β, IL-6, TNF-α, NF-κB, cyclooxygenase 1 (COX-1) and cyclooxygenase 2 (COX-2) [5,10,16,137].

The next subsections will describe the current knowledge about BCP action in different inflammation-related metabolic diseases.

#### 4.2.1. Obesity and Dyslipidemia

Obesity is a chronic metabolic disease characterized by excessive fat accumulation in adipose tissue [129]. The prevalence of obesity has increased globally over the last two decades [125], as a consequence of our evolutionary history [143]. Obesity is a multifactorial disease, caused by interactions between environment, lifestyle and genetics. Comprehension of these multiple factors is still ongoing and necessary for developing efficient strategies for obesity prevention and treatment [122].

According to the World Health Organization’s report dated April 2020, worldwide obesity has nearly tripled since 1975. In 2016, more than 1.9 billion adults were overweight; of these, over 650 million were obese. Moreover, in 2019 an estimated 38.2 million children under the age of 5 were overweight or obese. Once considered a problem only for high-income countries, overweight and obesity are now on the rise in low- and middle-income countries, particularly in urban settings. In Africa, the number of overweight children under 5 has increased by nearly 24% percent since 2000. Almost half of the children under 5 who were overweight or obese in 2019 lived in Asia [144].

Obesity correlates with cardiovascular risk, since it is associated with increased fasting plasma triglycerides and low density lipoprotein (LDL) cholesterol, low levels of high density lipoprotein (HDL) cholesterol, elevated blood glucose and insulin levels, and high blood pressure [145]. Specifically, the elevation of lipids in the blood is a condition called dyslipidemia, nowadays considered the major risk factor for the development of atherosclerotic disease and subsequent cardiovascular disease [146,147].

As mentioned before, obesity and overweight are characterized by chronic, low-grade inflammation, which perpetuates the disease and is associated with multiple complications [148], including the increase of inflammatory markers in liver, adipose tissue, skeletal muscle, pancreatic islets, and brain. Although the relationships between these events in rodents or obese humans remain poorly understood [94], in the last few years it has been suggested that adipocyte dysfunction is the trigger of obesity-related inflammation [149].

Several studies have been focused on possible strategies to treat obesity. Among the approaches based on natural compounds and bioactive molecules, the use of BCP has been tested both *in vivo* and *in vitro*. In this regard, we recently demonstrated an *in vitro* anti-obesogenic effect of 1 nM–10 μM BCP extracted from *Piper nigrum* and showed that it was able to reduce intracellular triglycerides accumulation without interfering with adipocyte number in the murine 3T3-L1 adipocytes [133]. The same *in vitro* model was already used to prove that dietary 5 or 10 μM BCP inhibits lipid accumulation in adipocytes and 0.15%–0.3% BCP dietary supplementation suppresses *in vivo* body weight gain and fasting blood glucose levels in high fat diet (HFD)-fed mice [134].

Another *in vitro* study tested the effects of 0.1–100 μM BCP on osteoblastic mineralization, osteoclastogenesis and adipogenesis in a bone marrow mesenchymal stem cells (MSC) model, demonstrating that BCP significantly suppresses the differentiation of bone marrow cells into adipocytes in a dose-dependent manner [85]. In an *in vivo* experiment on Wistar rats fed with high fat/fructose diet (HFFD), the effect of 30 mg/Kg b.w. /day BCP administration for 4 weeks by oral gavage on diet-induced dyslipidemia and inflammation was assessed [10]. In this study, BCP was able to decrease the visceral fat index, total cholesterol, LDL, very low density lipoprotein (VLDL), and pro-inflammatory cytokines (TNF-α and NF-κB). These effects were reversed by treatment with CB2 and PPAR-γ antagonists, suggesting that BCP activity is mediated by direct binding to CB2 receptors and by the activation of PPAR-γ, possibly through a cross-talk between these two receptor systems [10].

The effect of BCP on hypercholesterolemia was tested in a rat model of Triton-induced hyperlipidemia; 30 mg/Kg b.w. BCP was found to reduce total cholesterol, triglycerides, and LDL cholesterol levels in hypercholesterolemic animals and to exert hypolipidemic effects via inhibition of the hepatic HMG-CoA reductase [126]. The hypocholesterolemic effect of 30 mg/Kg b.w. BCP was also demonstrated in rats fed with cholesterol and fat enriched diet (HCFD); a significant decrease in serum total cholesterol and LDL, and an increase in HDL levels were observed also in this case [135].

In a mouse model of nonalcoholic steatohepatitis, 0.02% and 0.2% BCP supplementation in the diet exerted an antioxidant action and reduced hepatic steatosis as well as liver inflammation and fibrosis [136].

#### 4.2.2. NAFLD and NASH

Liver diseases are among the major causes of illness worldwide, and are caused by viral infections, alcohol abuse, abnormal dietary fat ingestion. In particular, NAFLD and the consequent NASH are considered as hepatic manifestation of MetS and strictly correlate with insulin resistance, obesity, dyslipidemia, atherosclerosis, and hypertension [116,150,151]. NAFLD is characterized by excess accumulation of triglycerides in hepatocytes due to both increased ingestion of free fatty acids (FFAs) and *de novo* hepatic lipogenesis. The accumulation of lipids causes oxidative stress and inflammatory response leading to NASH, which may progress to cirrhosis and liver cancer [78,116,150].

There is multiple scientific evidence on the beneficial effects of BCP on NAFLD and NASH experimental models. Clove extract (5 μM) was demonstrated to potently suppress the palmitate-induced lipid accumulation in human HepG2 hepatocytes, used as a model of *in vitro* NAFLD [78]. The major active molecule was found to be BCP, exerting its effects by binding to CB2 receptors, with subsequent AMPK phosphorylation, which in turn led to the upregulation of the lipolytic enzyme adipose triglyceride lipase (ATGL) and the downregulation of the lipogenic enzyme fatty acid synthase (FAS). In another *in vitro* study on HepG2 cells, BCP stimulation (1, 10 or 100 µM) led to a significant reduction of intracellular triglycerides and an increase of hepatic FFA uptake and FFA oxidation, via a PPARα-dependent mechanism [23].

In hypercholesterolemic Wistar rats fed with high cholesterol and fat diet, 1 mL/Kg b.w. or 30 mg/Kg b.w. BCP administration correlated with decreased hepatomegaly, lower hepatic lipid accumulation and steatosis, and decreased aspartate aminotransferase (AST) and alanine aminotransferase (ALT) activities; the hypolipidemic effects were mediated through inhibition of the hepatic HMG-CoA reductase [126,135].

In mice fed with methionine- and choline-deficient diet (MCD) reproducing the histopathological features of human NASH, BCP administration (0.2% of total diet for 8 weeks) exerted beneficial effects against hepatic steatosis, liver damage and inflammation found in the development and progression of NASH [136].

#### 4.2.3. Diabetes

T2D is considered one of the most important chronic diseases with severe complications, responsible for elevated indexes of morbidity and mortality [152]. The combination of genetic factors associated with impaired insulin secretion, insulin resistance, environmental factors, including overeating, aging, obesity and lack of exercise, typically accounts for T2D [153]. Moreover, insulin resistance can be a consequence of a reduction of insulin receptors numbers or a failure in insulin-receptor binding or in glucose transportation into the cell by the glucose transporter GLUT4 [152]. Inflammatory pathways have been suggested as the underlying and unifying pathogenic mediators for obesity and diabetes mellitus [154]. Indeed, an increase in body weight results in a dysfunction of the adipose tissue, with a greater release of proinflammatory cytokines, such as IL-6 and TNF-α. These molecules, together with increased FFA, can alter insulin sensitivity by stimulating the phosphorylation of serine instead of tyrosine residues in insulin receptor substrate-1 (IRS-1), thereby preventing the activation of insulin signaling pathway and making tissues less responsive to its action until insulin resistance [152,155].

Both *in vivo* and *in vitro* studies suggested a potential impact BCP on glucose metabolism. In particular, by *in vitro* analysis in skeletal myotubes (C2C12 cells) we demonstrated that 1, 10 and 100 nM of BCP was as efficient as insulin in stimulating cellular glucose uptake [133]. Besides, BCP induced the translocation of the GLUT4 storage vesicles to the plasma membrane of C2C12 cells, a process that, mainly in skeletal muscle and in adipose tissue, is directly correlated with the ability to lower elevated blood glucose levels. Other studies demonstrated that BCP induces insulin secretion in rat insulinoma (RIN-5F cells) [131], and in mouse pancreatic β−(MIN) cell line, through activation of CB2 and small G proteins ADP ribosylation factor (Arf)6, Ras-related C3 botulinum toxin substrate (Rac)1 and Cell division control protein (Cdc) 42 [156].

In streptozotocin (STZ)-induced diabetic rats (40 mg/Kg b.w.), the oral administration of 200 mg/Kg b.w. BCP significantly decreased blood glucose levels and increased plasma insulin; BCP increased the activity of metabolic enzymes such as hexokinase, pyruvate kinase and glucose-6-phosphate dehydrogenase in liver, kidney and skeletal muscle; BCP also reversed the activity of gluconeogenic enzymes that are deficient in diabetic rats, proving that it could normalize carbohydrate metabolism by enhancing glucose utilization and decreasing hepatic glucose production. Immunohistochemical analysis of pancreas sections demonstrated that BCP treatment improved tissue structure and increased insulin-secreting cell number, suggesting an insulinotropic effect of BCP [138]. In another study, oral administration of 200 mg/Kg b.w. BCP to STZ-induced diabetic rats significantly improved the levels of antioxidant enzymes, decreased lipid peroxidative markers in plasma and pancreatic tissues and reversed proinflammatory cytokines (TNF-α and IL-6) to near normal levels, thus indicating a relevant anti-inflammatory role of BCP in preventing diabetes-induced oxidative stress and associated complications [139].

An *ex vivo* and *in vivo* study in diabetic rats, also suggested an antidiabetic effect of BCP alone or in combination with dietary supplementation of L-arginine, that displays pancreatic β cell regenerative effects through nitric oxide (NO) modulation [131].

The protective effects of 30 mg/Kg b.w. BCP treatment on metabolic alterations were also highlighted in rats receiving a high fat/fructose diet (HFFD) that causes insulin resistance and obesity, where BCP administration prevented HFFD-induced elevation of adipose-index, hyperglycemia and hyperinsulinemia, acting through the activation of CB2 receptors [10].

#### 4.2.4. Cardiovascular Disorders

Metabolic dysfunctions are strictly related to cardiovascular inflammatory responses [157]. In particular, diet-induced disorders augment atherogenic complications and exacerbate vascular inflammation, leading to several detrimental outcomes as vascular wall thickness, platelet activation and predisposition to thrombosis [158,159]. This atherosclerotic process underlies ischemic diseases, among which myocardial infarction, the leading cause of death worldwide. Current established therapeutic options to prevent and treat atherosclerosis include inhibitors of cholesterol synthesis (statins), inhibitors of fat breakdown in adipose tissue (niacin), inhibitors of platelet aggregation (aspirin) and antihypertensive drugs (β-blockers, renin-angiotensin system inhibitors) [160].

Among new experimental strategies, the use of plant-derived bioactive compounds, in combination with drugs or used as preventive intervention from early life, represents a promising approach. Anti-atherosclerotic properties have been shown by several bioactive compounds, such as Omega-3 fatty acids, phytosterols, phenolic compounds [161], and, very recently, BCP. In particular the role of BCP in the regulation of the inflammatory cascade has been investigated in order to evaluate its use as a therapeutic agent against cardiovascular damage. In this regard, rats fed with a high fat/high fructose diet showed an ameliorated lipid profile (higher levels of HDL and lower levels of triglycerides, total cholesterol, LDL and VLDL) when supplemented with BCP. Furthermore, 30 mg/Kg b.w. BCP induced a reduction in pro-inflammatory cytokines TNF-α and NF-kB and no expression of adhesion molecule vascular cell adhesion molecule (VCAM)-1 in the aorta, underlining a positive effect of the molecule against atherosclerotic burden [10]. Furthermore, oral administration of BCP to hypercholesterolemic rats reduces atherogenic index and coronary risk index, preventing cardiovascular damage [10,162].

Occlusion of vascular wall could be a possible exacerbation of atherosclerosis, thus causing ischemic tissue damage with improper tissue blood supply. When the heart is involved in prolonged ischemic condition, myocardial infarction can develop, thus causing loss of cardiac cells and impaired organ function. Younis and colleagues showed the protective role of 100 or 200 mg/Kg b.w. BCP against isoproterenol-induced myocardial infarction, underlining the reduction of the inflammatory response induced by the treatment through a CB2-independent pathway. Indeed, orally administered BCP inactivated the heat shock protein (HSP)-60/Toll-like Receptor (TLR)/ Myeloid differentiation primary response (MyD) 88/NF-kB pathway thus protecting the heart from pro-inflammatory cytokines and chemokines rising and reducing infarct size [140].

Among drug-mediated cardiotoxic inflammation, the deleterious effect of doxorubicin, an anthracycline used in chemotherapy, has been underlined. Doxorubicin treatment induced the expression of NF-kB and activation of pro-inflammatory cytokines and chemokines that are involved in the progression of the inflammatory response in the cardiac tissue [163]. Intraperitoneal injection of 25–100 mg/Kg b.w. BCP showed protective effects against doxorubicin toxicity, in fact, it reduced the expression of NF-kB, TNF-α, IL-1β and IL-6, and down-regulated COX-2 and iNOS, thus attenuating inflammatory response in the myocardium without altering the antitumor effect of the drug and suggesting the possible use of BCP to prevent cardiac damage induced by doxorubicin [141,142].

### 4.3. Activity of BCP in Pain and Other Nervous System Disorders

Acute and especially chronic pain is a serious social burden and it has been estimated that around 10% of population worldwide suffers from long-lasting pain [164]. The neuroprotective role of cannabinoids against pain and neurodegenerative diseases has been extensively demonstrated [165]. Moreover, the endocannabinoid system seems to play an important role on inflammation and nociception with analgesic effects in numerous pain conditions, frequently in hyperalgesic and inflammatory states [165]. In inflammatory hyperalgesia, CB2 receptors localized on mast and immune cells could possibly achieve pain inhibition by the reduction of prostanoids or cytokines release, which are responsible for peripheral nociceptor sensitization [7]. This evidence is of particular relevance, since, as we already mentioned, CB2 receptors can be activated by BCP binding. Indeed, several studies demonstrated the efficacy of BCP to treat neuropathies and pain [21,73].

Due to its lipophilicity, BCP easily penetrates cell membranes, while still presenting good oral bioavailability and a very large therapeutic window, with an oral 50% lethal dose (LD_50_) of more than 5000 mg/kg in rats [7]. Significant and dose-dependent antinociceptive response was produced by BCP without the presence of gastric damage [166]. Antiallodynic actions of BCP are exerted only through activation of local peripheral CB2 [7]. In neuropathic pain models, BCP reduced spinal neuroinflammation and the oral administration was more effective than the subcutaneously injected synthetic CB2 agonist JWH-133. Thus, BCP may be highly effective in the treatment of long-lasting, debilitating pain states [167]. BCP also prevents nucleoside reverse transcriptase inhibitors-induced mechanical allodynia, possibly via reducing the inflammatory response, and attenuates mechanical allodynia through CB2 receptor activation [73]. BCP induces decrement in expression of COX-2 and iNOS, which could suppress NF-κB activation and as a consequence promote analgesia [168]. Recently, BCP was found to attenuate mechanical allodynia induced by paclitaxel, a drug used in chemotherapy, in a CB2-dependent manner. Moreover, BCP was able to attenuate the development of paclitaxel-induced peripheral neuropathy by reducing mitogen-activated protein kinases (p38MAPK) and NF-kB activation and increased ionized calcium-binding adaptor molecule-1 (Iba-1) and IL-1β immunoreactivity promoted by paclitaxel [169].

Interestingly, BCP can diminish acute and chronic pain not only through the endocannabinoid system, but also through the opioid system [21]. This latter mechanism involves the participation of benzodiazepine and serotonin 1A (5-HT1A) receptors, as well as nitric oxide [170]. BCP is able to indirectly activate the opioid system through β-endorphin release, which in turn activates *μ*-opioid receptors on primary afferent neurons [166]. The role of the opioid system is further demonstrated by blockade with naloxone, resulting in the abolishment of BCP analgesic effects in acute and chronic pain models [171].

The degeneration of axons is a critical event in many neurodegenerative conditions including stroke, glaucoma, motor neuropathies, amyotrophic lateral sclerosis (ALS), Alzheimer’s, Parkinson’s and Huntington’s diseases [172,173]. BCP could have a beneficial role in inducing neuritogenesis through the activation of tropomyosin receptor kinase A (TrkA) receptors by a mechanism independent of nerve growth factor (NGF) or cannabinoid receptors [5].

Parkinson’s disease (PD) is a long-term neurodegenerative disorder characterized by progressive dopaminergic neurons loss in the substantia nigra pars compacta (SNc). Treatment of mice with BCP rescued dopaminergic neurons and decreased microglia and astrocyte activation, as evidenced by reduced levels of Iba-1 and glial fibrillary acidic protein (GFAP) expression [6]. BCP, in addition to attenuation of pro-inflammatory cytokines and inflammatory mediators such as COX-2 and iNOS, also restored antioxidant enzymes and inhibited lipid peroxidation as well as glutathione depletion [174]. BCP acts via multiple neuroprotective mechanisms in murine models, thus it may be viewed as a potential treatment and/or preventative agent for PD [175].

Administration of BCP protects against cerebral ischemic injury in rats and reduces astrogliosis and microglial activation in a transgenic mouse model of Alzheimer’s disease [89].

A beneficial effect of BCP was also found for multiple sclerosis, also known as encephalomyelitis disseminata, the most common inflammatory and demyelinating autoimmune disease of the central nervous system. BCP reduced the clinical score and severity of experimental autoimmune encephalomyelitis and inhibited H_2_O_2_, NO, TNF-α, interferon-γ (IFN-γ), and IL-17 production. Moreover, BCP treatment significantly reduced the numbers of inflammatory infiltrates and attenuated neurological damages in the CNS of experimental autoimmune encephalomyelitis mice [176].

Epilepsy is a neurological disease, and recurrent epileptic seizures and behavioral comorbidities such as depression, anxiety, psychosis, and cognitive deficits largely affect the quality of life of the patients with epilepsy and their families [177]. BCP was found to display anticonvulsant activity against seizures induced by pentylenetetrazole in mice. Since no adverse effects were observed when BCP was administered at the concentration of 100 mg/kg b.w., and because of the lack of genotoxicity [178], this compound was considered a potential new anticonvulsant drugs [179]. Moreover, BCP was clinically useful as an adjunct treatment against seizure spread and status epilepticus and concomitant oxidative stress, neurotoxicity and cognitive impairments [180].

**Figure 2:**
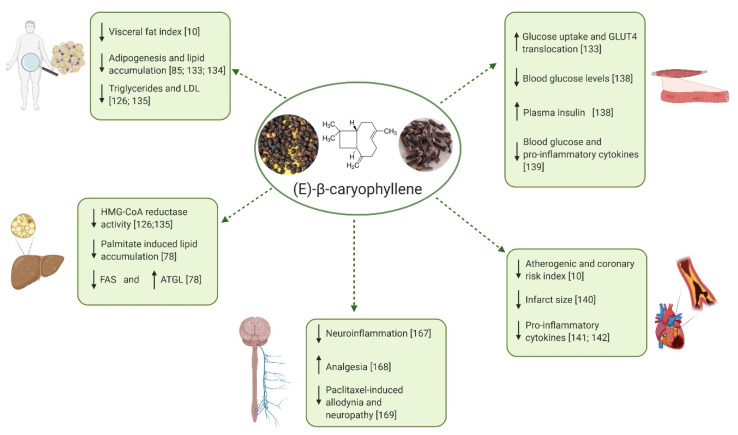
Positive effects of BCP on metabolic and neurological disorders. Scheme created with BioRender.com. ↓: reduction; ↑: increase; LDL: Low density lipoproteins; HMG-CoA: Hydroxy methylglutaryl-Coenzyme A; FAS: fatty acid synthase; ATGL: adipose triglyceride lipase; GLUT4: glucose transporter 4.

As a summary of the experimental evidence reported in this review, Figure 2 schematizes the major currently known positive effects of BCP on metabolic and neurological disorders.

## 5. Conclusions

The bicyclic sesquiterpene BCP is a natural compound widely present in the plant kingdom and obtained in high concentrations from the essential oils of several plants. As a secondary metabolite, particularly present in both vegetative and reproductive parts, BCP is primarily involved in plant defense and attraction. Recent studies outlined a protective role of BCP also in animal cells, underlining its beneficial effects against many diseases. These results have been summarized in previous reviews; the present review specifically focused on BCP action on diseases characterized by chronic inflammation.

Chronic inflammation is a common theme of many metabolic and neurologic disorders. The definition of new therapeutic approaches based on natural compounds could represent a promising way to complement, or even replace, currently administered drugs. The data accumulated so far both in *in vivo* and *in vitro* studies show that BCP is a good candidate in the treatment of chronic inflammation due to its specific molecular targets and very low toxicity. In fact, it is now widely accepted that this sesquiterpene acts on several molecular pathways implicated in the generation of inflammatory states and is able to reduce several pro-inflammatory mediators, including IL-1β, IL-6, TNF-α, NF-κB. The molecular mechanisms underlying BCP effects are only beginning to be unraveled. The available data suggest that BCP is able to exert its potent anti-inflammatory effects through multiple mechanisms mostly initiated by the binding of BCP to CB2 receptors. Subsequent steps likely depend on the cell type and grade of the inflammatory state. Recently, BCP activation of PPARs, a class of nuclear receptors involved both in metabolic and inflammatory responses, has been clearly demonstrated. Direct binding of BCP has been shown only for PPARα, while available data suggest CB2-mediated PPARγ activation. Further clarification of the molecular details involved in this receptor-cross-talk will strengthen the possible therapeutic use of BCP.

Although further studies are needed to better define the systemic effects of BCP in animals, the promising results obtained so far in preclinical studies on models of metabolic and neurologic disorders strongly suggest that BCP constitute an attractive molecule for the treatment of diseases characterized by chronic inflammation.

## Figures and Tables

**Table 1:** Evidence of a role of BCP on metabolic diseases.

Disease	Main Metabolic Effect	Experimental Model	BCP Administration	References
Obesity and dyslipidemia	Decrease of visceral fat index. LDL and VLDL	Wistar rats fed with HFFD	30 mg/Kg b.w./day for 4 weeks by oral gavage	[10]
	Inhibition of adipogenesis	Bone marrow cells	0.1–100 μM for 3–4 days in differentiation medium	[85]
	Inhibition of lipid accumulation	Preadipocytes (3T3-L1 cells)	1 nM–10 μM for 9 days in differentiation medium	[133]
			5 or 10 μM for 6 days in differentiation medium	[134]
	Suppression of body weight gain	HFD-fed C57BL/6N mice	0.15% or 0.3% supplemented diets for 16 weeks	[134]
			0.02% or 0.2% supplemented diets for 4 and 8 weeks	[136]
	Reduction of total cholesterol, triglycerides, and LDL cholesterol levels	Hypercholesterolemic Wistar rats	1 mL/Kg b.w. for 3 days by oral gavage	[126]
			30 mg/Kg b.w./day for 4 weeks by oral gavage	[135]
Hepatic steatosis	Decrease of hepatic HMG-CoA reductase activity	Hypercholesterolemic Wistar rats	1 mL/Kg b.w. for 3 days by oral gavage	[126]
			30 mg/Kg b.w./day for 4 weeks by oral gavage	[135]
	Inhibition of palmitate-inducible lipid accumulation Downregulation of FAS and upregulation of ATGL Reduction of triglycerides. increase of FFA uptake and FFA oxidation	Human hepatocyte cell line (HepG2)	5 μM for 24h in serum free medium	[78]
			1, 10 or 100 μM for 24h	[23]
T2D	Increase of glucose uptake and GLUT4 translocation	Skeletal myotubes (C2C12 cells)	1, 10, 100 nM for 30 min in glucose and serum free medium	[133]
	Decrease of blood glucose levels and proinflammatory cytokines levels Increase of plasma insulin	Streptozotocin-Induced Diabetic rats	200 mg/Kg b.w. for 45 days by oral gavage	[138,139]
	Decrease of fasting blood glucose and fasting insulin	Wistar rats fed with a HFFD	30 mg/Kg b.w./day for 4 weeks by oral gavage	[10]
Cardiovascular disorders	Reduction of atherogenic and coronary risk index	Hypercholesterolemic Wistar rats	30 mg/Kg b.w./day for 4 weeks by oral gavage	[10]
	Protective role against isoproterenol-induced myocardial infarction	Male Sprague–Dawley rats	100 or 200 mg/Kg b.w/day for 21 days orally	[140]
	Protective effect against Doxorubicin-induced inflammation in the myocardium	Male Wistar Rats	25, 50, 100 mg/Kg b.w. for 5 days by intraperitoneal injection	[141]
			25 mg/Kg b.w. for 6 days a week for 5 weeks by intraperitoneal injection	[142]

HFFD: high fat/fructose diet; HFD: high fat diet; LDL: low density lipoprotein; HMG-CoA: Hydroxy methylglutaryl-Coenzyme A; FAS: fatty acid synthase; ATGL: adipose triglyceride lipase; GLUT4: glucose transporter 4; VLDL: very low density lipoprotein; FFA: free fatty acids.
